# A Family Affair: Addressing the Challenges of Factor H and the Related Proteins

**DOI:** 10.3389/fimmu.2021.660194

**Published:** 2021-03-30

**Authors:** Felix Poppelaars, Elena Goicoechea de Jorge, Ilse Jongerius, Antje J. Baeumner, Mark-Steven Steiner, Mihály Józsi, Erik J. M. Toonen, Diana Pauly, the SciFiMed consortium

**Affiliations:** ^1^Department of Internal Medicine, Division of Nephrology, University Medical Center Groningen, University of Groningen, Groningen, Netherlands; ^2^Department of Immunology, Faculty of Medicine, Complutense University and Research Institute Hospital 12 de Octubre (imas12), Madrid, Spain; ^3^Department of Immunopathology, Sanquin Research and Landsteiner Laboratory of the Academic Medical Centre, University of Amsterdam, Amsterdam, Netherlands; ^4^Department of Pediatric Immunology, Rheumatology, and Infectious Diseases, Emma Children’s Hospital, Amsterdam University Medical Centre, Amsterdam, Netherlands; ^5^Institute of Analytical Chemistry, Chemo-and Biosensors, Faculty of Chemistry and Pharmacy, University of Regensburg, Regensburg, Germany; ^6^Microcoat Biotechnologie GmbH, Bernried am Starnberger See, Germany; ^7^Department of Immunology, ELTE Eötvös Loránd University, Budapest, Hungary; ^8^MTA-ELTE Complement Research Group, Eötvös Loránd Research Network (ELKH), Department of Immunology, ELTE Eötvös Loránd University, Budapest, Hungary; ^9^R&D Department, Hycult Biotech, Uden, Netherlands; ^10^Department of Ophthalmology, University Hospital Regensburg, Regensburg, Germany; ^11^Experimental Ophthalmology, University Marburg, Marburg, Germany

**Keywords:** complement system, factor H (FH), factor H-related protein, factor H-like protein 1, challenges - development directions

## Abstract

Inflammation is a common denominator of diseases. The complement system, an intrinsic part of the innate immune system, is a key driver of inflammation in numerous disorders. Recently, a family of proteins has been suggested to be of vital importance in conditions characterized by complement dysregulation: the human Factor H (FH) family. This group of proteins consists of FH, Factor H-like protein 1 and five Factor H-related proteins. The FH family has been linked to infectious, vascular, eye, kidney and autoimmune diseases. In contrast to FH, the functions of the other highly homologous proteins are largely unknown and, hence, their role in the different disease-specific pathogenic mechanisms remains elusive. In this perspective review, we address the major challenges ahead in this emerging area, including 1) the controversies about the functional roles of the FH protein family, 2) the discrepancies in quantification of the FH protein family, 3) the unmet needs for validated tools and 4) limitations of animal models. Next, we also discuss the opportunities that exist for the immunology community. A strong multidisciplinary approach is required to solve these obstacles and is only possible through interdisciplinary collaboration between biologists, chemists, geneticists and physicians. We position this review in light of our own perspective, as principal investigators of the SciFiMed Consortium, a consortium aiming to create a comprehensive analytical system for the quantitative and functional assessment of the entire FH protein family.

“When you can measure what you are speaking about, and express it in numbers, you know something about it”

– William Thomson, 1st Baron Kelvin

## Introduction: The Factor H Protein Family

The complement system forms a major arm of innate immunity and is of importance to fight invading pathogens (1). It consists of over 50 proteins that activate each other in a fixed order *via* three distinct pathways; the classical (CP), lectin (LP) and alternative pathway (AP), which all lead to cleavage of C3 and C5. This results in labeling of pathogens with C3b, attraction of immune cells *via* the anaphylatoxins C3a and C5a, and formation of the membrane attack complex [reviewed in (2)]. While the complement system is traditionally seen as a plasma system, recent studies also describe its importance locally, perhaps even inside cells (3). In health, the complement system is tightly regulated to prevent unwanted activation, inflammation and tissue damage. It has long been known that complement dysregulation contributes to various inflammatory and autoimmune diseases (4–6). A number of membrane-bound and fluid phase regulators ensure that the complement system is well-controlled (reviewed in (7)). Here, we will focus on the main regulator of the alternative pathway, namely Factor H (FH). FH can distinguish between self and non-self, and prevents complement activation both on cellular surfaces and in the circulation (8). More specifically, FH can function as a co-factor for Factor I (FI)-mediated proteolysis of C3b into iC3b, a molecule that cannot further propagate pathway activation. FH can also compete with Factor B (FB) to inhibit formation of the C3(H_2_O)B fluid phase tickover complex. In addition, FH promotes the decay of existing C3bBb-complexes (i.e., the C3-convertase), as well as the C4bC2aC3b and C3bBbC3b-complexes (i.e., the C5 convertases). FH is composed of 20 repetitive units, called complement control protein (CCP) domains, in a “beads on a string” configuration. The CCPs are ~65 amino acids in length and contain two invariant disulfide bonds. The FH N-terminal (CCPs 1–4) is important for decay accelerating activity and co-factor activity, while the internal region (CCPs 6–8) and the C-terminal (CCPs 19–20) are needed for host/ligand recognition and thus also for complement regulation on host surfaces (9–11). The human gene for FH is located on chromosome 1 within the Regulators of Complement Activation (RCA) gene cluster. The RCA gene cluster contains more than sixty genes and includes a ~700 Kb region in which FH as well as the Factor H-Related (FHR) proteins are encoded (described below). The complement FHR genes (*CFHR*) contain several repeating regions believed to have resulted from large genomic duplication events leading to the production of FHR proteins with partly similar domains to FH (12).

FH like-1 (FHL-1) is an alternatively-spliced version of FH and shares the first 7 CCP domains of FH before terminating with a unique four amino acid C-terminal tail. FHL-1 contains the C3b binding and regulatory domains of FH and thereby retains the regulatory function of FH. Also, since FHL-1 contains the CCP domains 6–7 of FH, it is assumed that FHL-1 shares some of the FH ligands and the ability to regulate complement on certain surfaces (13–15). Indeed, FHL-1 has been shown to bind similar ligands as FH such as C-reactive protein (CRP), pentraxin 3, heparin and malondialdehyde epitopes (16–18). Nevertheless, clear differences exist between these two proteins such as the extra binding domains in FH (CCP 8–20), the distinctive three-dimensional conformation of both proteins, and the unique C-terminus of FHL-1. This suggests that FH and FHL-1 also bind to distinct ligands expressed in certain tissues. Moreover, it has been implied that FHL-1 has a local and tissue specific role instead of a systemic function like FH (13).

Humans also have five FHR proteins; FHR-1, FHR-2, FHR-3, FHR-4 and FHR-5, whose functions are poorly characterized (described in more detail in (8)). Yet, their importance is shown by the causal link between genetic alterations in *CFHR* and various diseases (i.e., IgA nephropathy (IgAN) (19–23), age-related macular degeneration (AMD) (24–28), invasive meningococcal disease (29–31), atypical hemolytic uremic syndrome (aHUS) (32) and C3 glomerulopathy (C3G) (33). All FHR proteins share a high degree of similarity with FH in their N-terminus (varying between 36 and 94%) and their C-terminus (varying between 36 and 100%) (34). Notably, the N-terminus of the FHR proteins resembles CCPs 6–8 of FH, while the C-terminus is similar to CCPs 19–20 of FH. FHR-5 is an exception to this, since FHR-5 shares homology to CCPs 6–7 as well as CCPs 10–14 and CCPs 19–20 of FH. The homology of the FHR proteins to the surface recognition domains of FH enables these proteins to bind similar ligands on surfaces including heparin and C3 activation fragments such as C3b or C3d (32). However, since all FHR proteins lack the domains of FH responsible for the regulatory activity, the FHR proteins will, unlike FH, most likely not provide protection to these surfaces against complement attack. The current belief is, therefore, that the FHR proteins antagonize the ability of FH to regulate complement activation (35). Furthermore, some FHR proteins can form dimers. FHR-1, FHR-2, and FHR-5 contain a dimerization motif in their N-terminal domains, while in FHR-3 and FHR-4 this motif is missing. This would enable FHR-1, FHR-2, and FHR-5 to form both homodimers and heterodimers. Accordingly, structural and sequence analyses suggested that, in addition to homodimers, FHR-1 can form heterodimers with FHR-2 and FHR-5, while FHR-2/FHR-5 heterodimers would only be formed in sera partially or totally deficient in FHR-1 (36). However, recently, another study suggested that only four dimers occur in circulation: homodimers of FHR-1, FHR-2, and FHR-5, as well as FHR-1/FHR-2 heterodimers (37). Further studies are therefore needed to confirm the exact nature of the dimer composition, as well as the precise function of these dimers.

## Brief Description of the History

In hindsight, the earliest publication about the FH family was in 1965, when Nilsson and Muller-Eberhard initially isolated FH from human serum and identified this novel protein as β1H globulin (38). Yet, it wasn’t until 1976 that two groups independently of each other discovered the C3b inhibitory activity of FH as well as its regulatory activity on the C3-convertase (39–41). In 1983, regulation of C5 convertases by FH was first described (42). Finally, in 1988, the genetic code of FH and its amino acid sequence were identified (43). This discovery was essential to uncover the structure of FH as discussed above. At around the same time of this breakthrough, Schwaeble *et al*. demonstrated the expression of an additional smaller truncated form of FH in the human liver, which we now know as FHL-1 (44). In 1989, the same group demonstrated that FHL-1 had FI-cofactor activity (45). In the end, in 1991, FH and FHL-1 were shown to be derived from the same gene by a process of alternative splicing (46, 47).

One of the earliest mentions on any of the FHR proteins was in a paper describing the isolation of murine FHR proteins by Vik et al. in 1990 (48). Due to the extensive number of large genomic duplications between the exons of *CFH* and the *CFHR* genes, determining the genomic positions of the human *CFHR* genes was challenging and was performed throughout the early to mid-1990’s. In 1991 and 1992, mRNA transcripts encoding for FHR-1, FHR-2 and FHR-3 were revealed (47, 49–51). Expression of FHRs on protein level were characterized and described soon after (44, 50, 52). The position of *CFHR2* was the first of the FHR proteins to be determined when it was identified within the region between *CFH* and *Factor XIII* (53). In the next years, the other three *CFHR* genes were mapped within the RCA cluster between *CFH* and *CFHR2* (54). However, due to the high sequence resemblances between these genes, the determination of their exact positioning was not possible. The last *CFHR* gene discovered was for FHR-5, which was first described at protein level in 2001 in studies of immune-complex-mediated kidney diseases (55, 56). Finally, the genetic location of *CFHR5* was determined using fluorescence *in situ* hybridization (FISH), radiation hybrid mapping and BLAST alignment (57). Ultimately, it wasn’t until 2002 that the genomic segment containing the *CFH* and *CFHR* gene family was confirmed and to have the gene positions from centromere to telomere: *CFH*, *CFHR3*, *CFHR1*, *CFHR4*, *CFHR2*, *CFHR5* (a schematic overview of the genomic organization of the *CFH* gene family is provided in (35)). Furthermore, nowadays other forms of the FH protein family have also been described, namely the alternatively spliced forms of *CFHR4* named FHR-4A and FHR-4B leading to a total of 8 proteins that are encoded by the human *CFH* and *CFHR* gene family (excluding the different glycosylation variants as well as the homo- and heterodimers) (an outline of the protein structure of the FH family is provided in (8, 35, 58).

## Factor H and the Related Proteins in Disease

In recent years, numerous conditions have been associated with mutations or polymorphisms in the *CFH* gene family [an overview is provided in (32, 35, 59)]. These findings support the notion that complement dysregulation due to alterations in the FH family are a unifying pathogenic feature of various pathologies. Deciphering the pathogenic mechanism by which this protein family leads to disease is crucial for establishing the right diagnosis and therapeutic interventions. Despite the association of genetic variants in the *CFH* gene family with diseases, little is known regarding the biological processes leading to inflammation and tissue injury. Understanding the molecular mechanisms behind these genetic associations is challenging and represents an area of intense research. Notably, the disease-associated genetic variants in the *CFHR1-5* are particularly difficult to interpret due to the lack of knowledge regarding the biological role of the FHRs. We and others have shown the existence of genotype-phenotype correlations between gene variants in the *CFH-CFHR1-5* and complement-mediated diseases demonstrating that, although the same genes associate with various diseases, the molecular mechanisms behind these associations are specific of each condition (15, 59–61). In this context, the studies performed with FH, the best-known member of the family, were the first ones to illustrate such genotype–phenotype correlations. Mutations causing plasma FH deficiencies were amongst the first *CFH* alterations described. When these *CFH* mutations are present in homozygosis or compound heterozygosis they lead to complete FH deficiency, which cause massive complement activation in fluid phase, and are commonly associated with C3G, a heterogeneous histopathological entity characterized by glomerular C3 accumulation (62). However, when the null *CFH* alleles are in heterozygosis they only lead to partial FH deficiencies, and are equally associated with C3G as well as other diseases such as aHUS, AMD and IgAN. In this scenario, the combination with other genetic, acquired and/or environmental risk factors that are specific for each disease determines the final phenotype outcome. Interestingly, missense mutations within the C-terminus of FH are prototypical of aHUS and cause an inappropriate regulation of complement on endothelial surfaces leading to tissue damage, but without altering complement regulation in the fluid phase (63–66).

In addition to *CFH*, strong associations between genetic modifications in *CFHR1-5* and pathologic outcome have also emerged (32). Amongst the disease-associated *CFHR1-5* variants, genomic rearrangements leading to deletions, duplications, or hybrid genes are the most remarkable and informative ones (59). Perhaps, the most extensively studied is the deletion of *CFHR3* and *CFHR1*, which has a variable allele frequency between 0–55% in various ethnic groups (67). Moreover, this common gene variant is associated with protection against the development of AMD and IgAN, while it increases the susceptibility for aHUS (due to anti-FH autoantibodies) and systemic lupus erythematosus (SLE) (21, 24, 68, 69). These different disease associations highlight the relevance of the context in defining the effect of the FHR-1 and FHR-3 deficiency, illustrating those situations where the promotion of complement activation by the FHR proteins may be detrimental (i.e., Bruch´s membrane and mesangium) or where it may be beneficial (i.e., apoptotic cells). Another captivating type of a genomic rearrangement of the *CFHRs* is the duplication of the dimerization domains in FHR-1, FHR-2 or FHR-5. These *CFHR* variations are exclusively associated with C3G (33, 70–73). In this case, the resulting proteins are gain-of-function mutants that present an increased avidity for their ligands (36). Hence, these mutant proteins are postulated to out–compete the binding of FH to C3b deposited on surfaces and impair complement regulation more efficiently than the corresponding wild-type proteins (32, 36).

Besides genetic modifications, systemic levels of FHRs may also be crucial in disease processes. In anti-neutrophil cytoplasmic antibody (ANCA)-associated glomerulonephritis, increased systemic levels of FHR-1 were found compared to healthy controls (74). Additionally, FHR-1 levels were shown to weakly correlate with lower renal function and the percentage of relapses increased with growing FHR-1 concentrations. In IgAN, two groups independently of each other reported that plasma levels of FHR-1 and the FHR-1/FH-ratio are elevated in these patients and associate with progressive disease (75, 76). In contrast, plasma FHR-5 and the FHR-5/FH-ratio were not associated with progressive disease (76). However, higher FHR-5 levels in IgAN did associate with histological disease severity. In aHUS, plasma FHR-3 levels were demonstrated to be elevated compared to controls even when taking the *CFHR3* genotype into account (77). Also, the aHUS-risk *CFH–CFHR3–CFHR1* haplotype was shown to be associated with increased plasma levels of FHR-3, suggesting that an imbalance between FH and FHR-3 concentration may predispose individuals to aHUS. Recently, increased systemic FHR-4 levels were shown to be strongly associated with AMD (28). This is the first time that FHR-4 has been associated with a disease. A genome-wide association study revealed that an intronic variant in *CFHR4* correlated with systemic complement activation in AMD patients and associated with an increased risk of AMD development (26). A follow-up study demonstrated that the *CFHR4* variant was associated with higher levels of FHR-4 (28). Moreover, circulating FHR-4 levels and the FHR-4/FH-ratio were demonstrated to be elevated in AMD compared to controls, and the protein co-localized with complement activation products in choriocapillaris beneath the retina.

In addition to autoimmune diseases, the FH family has also been known to be involved in infections (78). Pathogens evade complement attack by recruiting complement regulators such as FH onto their surface, and it is suggested that the FHR proteins have evolved as decoys to reduce the amount of FH that is acquired by the microbes (35, 79). An illustrative example of this situation was described by Caesar et al., who showed that FHR-3 competes with FH for the binding of a FH-binding protein on *Neisseria meningitidis*, acting as a FH antagonist, which explains why the *CFH* haplotype 3, characterized by low FH and high FHR-3 plasma levels, is associated with lower susceptibility to meningococcal disease (78, 80). However, in contrast, the deletion of *CFHR3* and *CFHR1* was found to be associated with better survival in patients with bacterial meningitis (81). Altogether, this demonstrates the complex and multifaceted roles of the FH family in infections.

Altogether, the associations of the FH family with these diseases illustrate the relevance of the delicate balance between the different family members. Notably, the ratio between the levels of the regulator FH and the FHR proteins (i.e., FHR-1, FHR-3, FHR-4 and FHR-5) seems crucial in determining the outcome. Hence, either genetic or environmental factors altering the protein levels or the functionality of these proteins will have an impact on complement regulation and will define the susceptibility for the development of pathological conditions.

## Major Challenges

**Figure 1:**
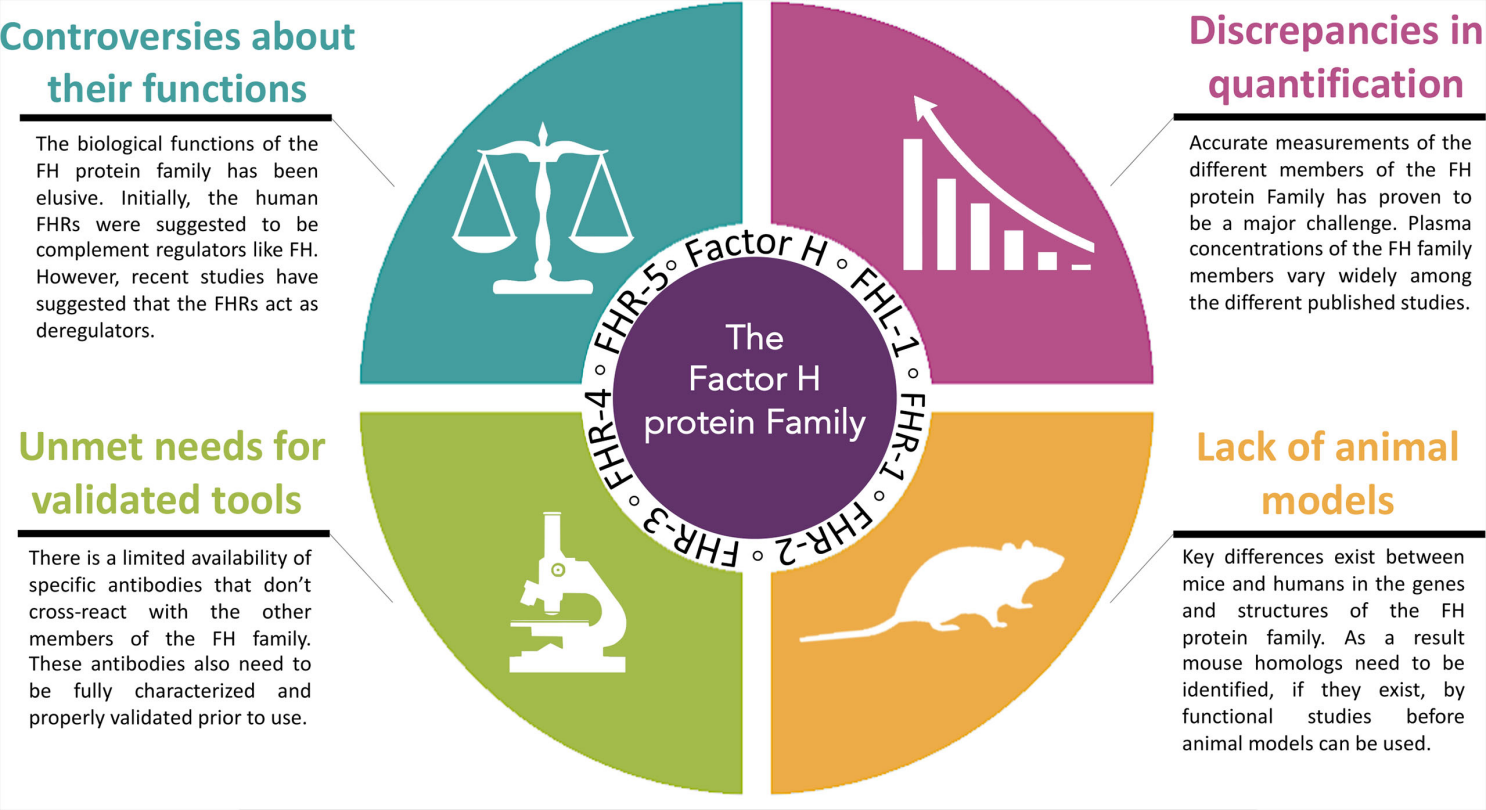
Four specific unmet challenges for the Factor H protein Family. The major challenges ahead in this emerging area are: 1. the controversies about the functional roles of the Factor H protein family, 2. the discrepancies in quantification of the Factor H protein family, 3. the unmet needs for validated tools and 4. limitations of animal models.

In the last few decades, major strides have been made in our understanding of the FH protein family. From these findings an appreciation has emerged of the vast complexity of this group of proteins as well as of the monoclonal antibodies developed to specifically detect the different members of the Factor H protein family. Unfortunately, we still face multiple unmet challenges. Many of these involve the need for reagents and models to better understand the function of FHR proteins in health and pathology. Here, we will discuss four specific unmet challenges that need to be resolved (**Figure 1**).

### Controversies in Functional Roles

The biological function of the FH protein family has been elusive. While it is clear that FH is a potent inhibitor of the complement system, and most data point towards a similar role for FHL-1, the functions of the more recently discovered FHR proteins are less well characterized and therefore remain uncertain. Initially, it was suggested that certain family members (e. g. FHR-1, FHR-3 and FHR-4) had no specific function or, at least, no essential function within the complement system. This rationale was largely based on the high frequency of *CFHR* gene deletions in the general population and the high homology among these proteins, suggesting some degree of functional redundancy. Instead, genetic studies revealed that alterations in the *CFHR* genes were indeed associated with pathology (described above), thereby providing the first piece of evidence that FHR proteins could be key pathogenic drivers of human disease. Since the pathogenesis of these diseases involve complement dysregulation, initial functional studies primarily focused on the potential regulatory functions of the FHR proteins. As a result, considerable controversy exists over whether the FHR proteins have complement regulatory capacity or not. Results from these earlier studies indicated that the FHR proteins primarily functioned as complement regulators at specific steps of the cascade, while this concept was contested by later studies (82). Specifically, first the interaction of FHR proteins with C3 was investigated by functional studies, as an indicator of their potential complement inhibiting capacity, since the related proteins were assumed to be functional analogues of FH. Indeed, FHR-3 and FHR-4B were able to bind to the C3d region of C3b (83). Yet, when FHR-3 and FHR-4 were first studied for their effect on Factor I-mediated C3b inactivation, direct co-factor activity was very weak and only detectable at very high, and non-physiological, concentrations (i.e., 400 µg/ml). Moreover, the addition of FHR-3 and FHR-4 also enhanced the inhibitory activity of FH. A later study demonstrated a small inhibitory effect for FHR-3 in the hemolysis assays using FH-depleted serum (84). The regulatory effect of FHR-3 was shown to be based on cofactor activity, although supraphysiological concentrations were once again used. In contrast, others could not show any significant cofactor activity for FHR-4, even at high concentrations (i.e., 650 µg/ml) (85). In this study, FHR-4 did slightly enhance the inhibitory activity of FH. Later, FHR-2 was shown to bind C3b and C3d (86). While no cofactor or decay accelerating activity was found for FHR-2, it was shown to inhibit the activity of the C3bBb-convertase. For FHR-5, weak cofactor activity and fluid phase C3-convertase inhibiting activity were reported, once again at very high concentrations (87). FHR-5 was also found to inhibit both the C5-convertases of the CP and AP in an artificial, bead-based *in vitro* model (88). In these latter assays, the effective FHR-5 concentrations were close to serum levels measured in samples from healthy donors and patients with glomerulonephritis (37, 76, 87, 89). In conformity, FHR-5 produced by glioblastoma cells was also shown to act as a co-factor of Factor I and inhibit terminal pathway activation, although solely at high concentrations (90). Likewise, inhibition at the C5 level and/or the terminal pathway has also been reported for FHR-1, FHR-2 and FHR-3 (84, 86, 91). Fittingly, in a mouse model of a neurological autoimmune disease, injection of FHR-1 expressing neural stem cells ameliorated brain injury. Human FHR-1 was shown to protect astrocytes from complement activation by inhibiting the formation of the membrane attack complex (92). However, others have not been able to find any significant inhibiting activity of FHR-1 on the terminal pathway (36, 93–95).

Despite these initial studies, it has been very difficult to reconcile the reported regulatory activities of FHR proteins with their structures, especially considering the lack of structural homology of the related proteins with the regulatory domains of FH. In recent years, accumulating data strongly indicated a role for the FHR proteins as promoters of complement activation that stands in contrast to the regulatory function of FH and FHL-1. The study by Hebecker and Józsi was the first to challenge the paradigm, demonstrating that, by binding C3b, FHR-4 in fact enhances alternative pathway activation (85), a mechanism which was also suggested previously by Närkiö-Mäkelä et al. (96). This property of FHR-4 was recently exploited to overcome complement resistance of HER-2 positive tumor cells by applying FHR-4 based immunoconjugates (97). Studies by Tortajada et al. and Goicoechea et al. soon followed and described another mechanism, namely de-regulation by FHRs through competition with FH. FHR-1, FHR-2 and FHR-5 were shown to form homo- and hetero-oligomeric complexes, while a C3G-associated mutation in FHR-1 resulted in the duplication of the dimerization domain leading to the formation of unusually large multimeric FHR complexes that exhibited increased avidity for C3 activation fragments (36, 71). Similarly, in IgAN, elevated levels of FHR proteins were shown to be associated with enhanced complement activation, while the absence of FHR-1 and FHR-3 was shown to decrease complement activation in AMD. These functional roles are opposite to that proposed in prior studies but are entirely consistent with the FHR structures, wherein the homologies of FHR proteins with FH are in the surface ligand-binding sites. Moreover, these studies strongly suggest that FHR proteins compete with FH (and FHL-1) mediated inhibition and thereby antagonize this key regulator of the complement system. Overall, FHRs were indeed shown to enhance complement activation both directly and indirectly (i.e., *via* competing with FH), thus emerging as “regulators of the regulator” (34). Competition between FHRs and FH has been described for several binding ligands. FHR-1, FHR-3, FHR-4 and FHR-5 were all shown to compete with FH for binding to C3b, to variable extent. Some of these differential effects may be related to the different avidities also determined by homo- or heterodimerization of FHR-1 and FHR-5 (36, 80, 84, 91). In addition, FHR-5 has been shown to strongly inhibit the binding of FH to the pentraxins (i.e., CRP and PTX3), as well as to extracellular matrix and malondialdehyde-acetaldehyde epitopes (98, 99). Subsequently, FHR-5 enhanced AP activation on these ligands. For FHR-1, FHR-4 and FHR-5 it has been shown that, by binding C3b, they can serve as a platform for the assembly of a functionally active C3bBbP convertase, consequently enhancing activation of the AP (85, 95, 98). Furthermore, FHR-5 can also induce AP activating by the recruitment of properdin *via* the CCPs 1-2 (99). Interestingly, in addition to modulation of the AP, FHR-1 and FHR-4 were both shown to activate the CP as well through the binding of CRP (i.e., FHR-1 the monomeric form, and FHR-4 the native, pentameric CRP) (85, 95, 100). More recently, FHR-1 and FHR-5 were shown to compete with FH for binding to DNA and thus promote AP activation, as well as to modulate both AP and CP activation on the surface of necrotic cells *via* interactions with monomeric CRP and PTX3 (101).

In conclusion, while complement inhibiting activity for some of the FHR proteins was described, the reported inhibitory activities were typically weak. More recent studies suggest that FHR proteins represent pattern recognition molecules that promote rather than constrain complement activation (35). To resolve the controversy, these functions need to be studied further, using physiological concentrations, and confirmed by independent research groups. The reported discrepancies may be related to the various sources of recombinant proteins used in the studies. In addition, the proteins may display different, context-dependent activities. Besides the function of the FH-family in the regulation of the complement system, non-canonical functions such as regulating cellular responses were described for FH, FHR-1 and FHR-3 (discussed in detail elsewhere (102)). Losse *et al*. reported that FHR-1 can bind to neutrophils *via* complement receptor 3 (CD11b/CD18), thereby resulting in enhanced antimicrobial activity (103). Recently, FHR-1 was shown to activate the NLRP3 inflammasome *via* the EMR2 receptor on monocytes and by binding to necrotic cells (74). Furthermore, these mentioned controversies raise an additional important question: How does the FH-family regulate inflammation, and what are their ligands and are there FHR protein receptors? Future studies need to define specific and shared ligands among members of this protein family, as well as conditions under which physiological or pathological functions are displayed, or competition occurs.

### Discrepancies in Quantification

Accurate analysis of the FH protein family is of utmost importance for further deciphering of its function and role in complement-mediated diseases. Accurate information on physiological levels and the composition of the FH family members is also vital for functional studies, since supraphysiological levels can give misleading results. Yet, precise analysis of FH and other complement system components has proven to be challenging and systemic levels for FH family members vary widely among different studies (**Table 1**). These inconsistencies in levels are not only due to differences in sample type (plasma *vs* serum or different anticoagulants), storage (room temperature, 4°C *vs* -20°C or -80°C) and pre-analytical sample handling between studies, but also most likely caused by the use of different techniques (ELISA vs mass spectrometry), protocols and reagents (113). Another important explanation for the discrepancies in reported levels could be differences in characteristics of the blood donors. Age and gender have previously been demonstrated to significantly impact complement levels and functionality in the healthy population (114). Nevertheless, the impact of age and gender on the FH protein family has not been extensively studied. In healthy children, FHR-1, FHR-4A and FHR-5 levels were shown to be slightly lower in children compared to adults, but only FHR-5 levels were significantly associated with age (115). In addition, no gender differences were found. Levels of FH, FHR-2, and FHR-3 were similar to those found in adults (115). However, when corrected for genetic factors, an age-dependent increase of plasma levels of FH was seen for individuals aged 1 to 88 years (105). Furthermore, even when laboratories use the same technique, for instance ELISA, varying methods, reagents, calibrators and antibodies are used. Moreover, when antibodies are used, it is not always known whether these antibodies are truly specific for the target antigen or if cross-reactivity with other proteins may occur. Given that the FH protein family has a high degree of similarity in amino acid sequence, it is very well possible that antibodies against FH also cross-react with other FH family members. As a result, large discrepancies in levels for FH family members are observed between testing laboratories thereby hampering correct interpretation and hindering the comparison of results between studies. These inconsistencies in levels indicate the urgent need for well-characterized and standardized assays (116). Yet, validated and standardized assays for quantitative and functional analysis are not (widely) available for FH and its related proteins. Here, epitope mapping can be extremely valuable to predict (potential) cross-reactivity with other FH family members. The epitope location of a monoclonal antibody (mAb) can be determined using fragments consisting of CCP domains as previously described (111). It is important to note that in contrary to the FHR proteins, the functions of FH are well known and several functional assays for FH exist, some of which currently are being used in the clinic. FH-mediated decay-accelerating activity can be measured in both ELISA as well as surface plasmon resonance assays (117–119). The co-factor activity of FH can be determined in both fluid-phase as well as on the cell surface (120, 121). In addition, the full function of FH can be addressed on cell surfaces using cell-based assays (13, 117, 119).

**Table 1:** Previously published systemic levels of the Factor H protein family.

Protein	Genotype	Levels (µg ml^−1^)	N	Reference
**Total Factor H**	N.D.	400 ± 62	1004	(104)
	N.D.	319.9 ± 71.4	358	(105)
	N.D.	233.2 ± 56.7	63	(106)
	N.D.	232.7 ± 74.5	1514	(107)
	N.D.	152.5 (95%-CI: 123 - 190)	161	(76)
	No ΔCFHR1/3 1*ΔCFHR1/3 2*ΔCFHR1/3	156 ± 39 168 ± 49 176 ± 39	44 24 8	(75)
	N.D.	349.0 (95%-CI: 339 - 359) 304.7 (95%-CI: 297 - 312)	214 308	(28)
**Total FHL-1**	N.D.	47 ± 11.3	2	(108)
	N.D.	∼2.33 (or ∼0.04 μM)	3	(13)
**Total FHR-1**	1*CFHR1 2*CFHR1	61 ± 34 122 ± 26	24 44	(75)
	N.D.	94 [IQR: 70.5 – 119.6]	161	(76)
	N.D.	1.63 ± 0.04	344	(109)
	N.D.	26.5 ± 2.3	55	(74)
	N.D.	70 – 100	?	(91)
**FHR-1 homodimers**	1*CFHR1 2*CFHR1	4.88 ± 1.33 14.64 ± 3.04	36 77	(37)
**FHR-1/2 heterodimers**	1*CFHR1 2*CFHR1	5.01 ± 1.49 5.84 ± 2.41	36 77	(37)
**FHR-2 homodimers**	0*CFHR1 1*CFHR1 2*CFHR1	3.1 0.85 ± 0.41 0.65 ± 0.41	4 36 77	(37)
**Total FHR-2**	N.D.	3.64 ± 1.2	344	(109)
**Total FHR-3**	1*CFHR3 2*CFHR3	0.38 ± 0.23 0.83 ± 0.48	26 69	(77)
	2*CFHR3*A 2*CFHR3*B	0.55 ± 0.15 0.82 ± 0.08	16 4	(77)
	N.D.	1.06 ± 0.53	21	(110)
	N.D.	0.020 ± 0.001	344	(109)
**Total FHR-4**	N.D.	25.4 [IQR: 6.5 - 53.9]	11	(85)
	N.D.	2.42 ± 0.18	344	(109)
	N.D.	5.5 (95%-CI: 4.9 - 6.2) 6.0 (95%-CI: 5.6 - 6.3)	214 308	(28)
**FHR-4A**	N.D.	2.55 ± 1.46	129	(111)
**FHR-4B**	N.D.	Not detected	?	(111)
**Total FHR-5**	N.D.	5.5 [IQR: 3.4 – 10.1]	13	(89)
	N.D.	5.49 ± 1.55	344	(109)
	N.D.	2.46 [IQR: 1.79 – 3.67]	158	(76)
	N.D.	3.19 [IQR: 2.55 – 3.92]	153	(112)
**Homodimers FHR-5**	N.D.	1.66 ± 0.43	115	(37)

An overview of the published systemic levels of Factor H, Factor H-like 1 and the five Factor H-related proteins in healthy controls. Specific information is provided per study on the genetic background of the cohort, the number of subjects and whether they measured total levels, homo- or heterodimers. Data are presented as mean ± standard deviation (SD), mean with 95%-confidence interval (95%-CI) or median with interquartile range [IQR].

FH, Factor H; FHL-1. Factor H-like 1; FHR, Factor H-related protein; N.D., not determined.

In order to provide insight into the magnitude of the discrepancies in quantification of the FH protein family, we determined systemic FH levels in samples from healthy volunteers using seven commercially available ELISA’s (**Table 2**). Levels of FH were evaluated in samples that were collected, stored and handled exactly the same way. Next to those 10 samples, we also included 2 samples obtained from the Complement EQA Group. Both samples consist of a pool of serum samples derived from 5 healthy individuals. The Complement EQA Group committee aims to standardize complement analysis by providing calibrator materials and collects, evaluates and compares levels of complement components from different testing facilities (see also below). It should be noted that within the FH protein family, only quantitative analysis of serum levels of FH is included in the standardization activities of the Complement EQA Group. In 2009, this group was formally recognized and became part of the IUIS (International Union of Immunological Societies) Quality Assessment and Standardization Committee (https://iuis.org/committees/qas/) (122). We chose plasma-EDTA samples, since coagulation enzymes can also cleave complement components with subsequent generation of activation products (122). EDTA blocks the *in vitro* activation of the complement system by Mg^2+^ and Ca^2+^ chelation. Citrate-based anticoagulants are less useful (123). Moreover, heparin-plasma should not be used since multiple members from the FH family are heparin-binding proteins, hence heparin could interfere with the measurements. All assays were performed in parallel by the same operator according to the manufacturer’s protocols. In general, all %CV were ≤15%, indicating low variation.

**Table 2:** Human Factor H ELISA’s included in the comparison analysis.

Company	Name	Cat#	Lot#	Website
Abcam	Human FH ELISA	ab137975	GR3261729-8	www.abcam.com
Hycult Biotech	Complement FH, human, ELISA kit	HK342	28643K0420	www.hycultbiotech.com
LSBio	LSbio CFH	LS-F21748	189699	www.lsbio.com
Quidel	MicroVue FH EIA	A039	184358	www.quidel.com
R&Dsystems	CFH duoset	DY4779	P240815	www.rndsystems.com
Sanquin	Human FH ELISA	No info*	No info*	www.sanquin.nl
USCN	CFH ELISA kit	SEA635Hu	L200831651	www.uscnk.com

*The assay of Sanquin is only available as service. Samples can be sent to Sanquin for analysis.

**Figure 2:**
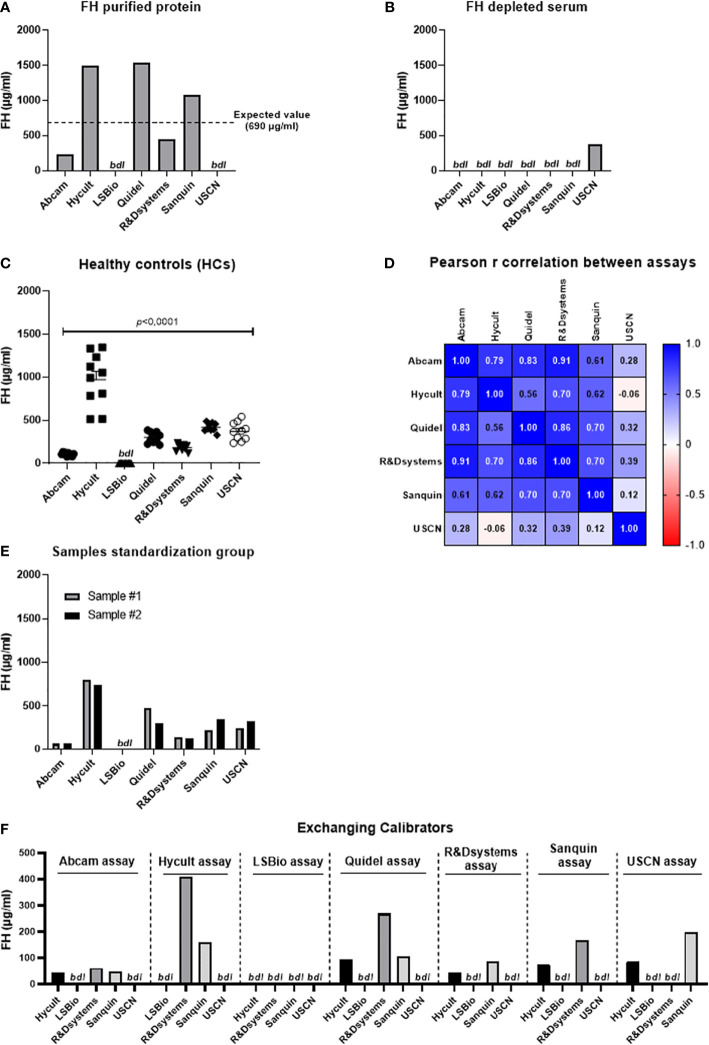
Assessment of Factor H levels in samples using seven different assays. **(A)** Assessment of Factor H (FH) purified protein in PBS (expected value is 690 µg/ml). **(B)** Assessment of FH levels in FH depleted serum. **(C)** Assessment of systemic FH levels in samples derived from healthy controls (n=10). Data are represented as mean ± SEM. Data were analyzed using the one-way ANOVA (Graphpad Prism 8.4.2, San Diego, CA, USA). A p-value <0.05 was considered significant. **(D)** Pearson r correlation coefficient. Pearson coefficients range from +1 to -1, with +1 representing a positive correlation of FH sample values between assays, -1 representing a negative correlation of FH sample values between assays, and 0 representing no relationship. No correlation could be calculated for the LSBio assay as no FH levels were obtained using this assay. **(E)** Assessment of FH levels in 2 serum pool samples obtained from the Complement EQA Group. **(F)** Assessment of calibrators as sample in each assay. All calibrators were exchanged between the seven assays except for the Abcam and Quidel calibrator as not enough calibrator was provided with these kits to be included in each assay as sample. FH, factor H; HCs, healthy controls; bdl, below detection limit.

First, human purified FH protein dissolved in PBS was measured in all seven assays (Cat# A137, Complement Technology Inc., TX, USA). None of the assays was able to ‘pinpoint’ this exact concentration (not corrected for the extinction coefficient). The assays from LSBio and USCN were not even able to detect purified FH protein in PBS (**Figure 2A**). Next, a FH depleted sample was measured as a negative control in all assays (Cat# A337, Complement Technology Inc., TX, USA). As expected, most assays did not detect FH in this sample. However, a FH concentration of 377 µg/ml was measured in the FH depleted sample using the USCN assay (**Figure 2B**). Subsequently, we assessed systemic FH levels in plasma-EDTA samples derived from 10 healthy controls. Results show large and significant differences in FH levels between the seven assays used (*P*<0.0001). No FH was detected with the LSBio assay (**Figure 2C**). In spite of these differences in absolute FH levels between the assays, moderate to high correlations were observed between the Abcam, Hycult, Quidel, R&Dsystems and Sanquin assays regarding the FH levels (**Figure 2D**). No correlation was observed between the USCN and the other assay. For the LSBio assay, no correlation could be calculated as no FH levels were detected with this assay. The FH levels in the serum pool samples obtained from the Complement EQA Group were comparable with the levels measured in the healthy control panel. Again, no FH was detected using the LSBio assay (**Figure 2E**). Lastly, calibrators were exchanged, except for the Abcam and Quidel ELISAs, as not enough calibrator was provided to be included in each assay as sample. Results show that the calibrators from Hycult, R&Dsystems and Sanquin were exchangeable, and yielded quantifiable and reliable levels. The calibrators from LSBio and USCN were not recognized in all other assays. In turn, the LSBio assay was not able to recognize/measure any of the calibrators except its ‘own’ calibrator. The USCN assay was able to recognize the calibrators from Hycult and Sanquin (**Figure 2F**). Overall, the assays from Abcam, Hycult, R&Dsystems and Sanquin were perfectly able to detect FH protein in the positive control and did not detect FH in the negative control. Additionally, the correlations between these assays were moderate to high. In contrast, the results obtained with the LSBio and the USCN assay suggest that these assays are not able to assess FH levels in samples in a reliable manner. Nevertheless, even in the reliable assays, FH levels obtained in the same sample set vary greatly. Given these discrepancies, we can conclude that the absolute FH levels determined with these assays, are probably not correct. Considering the lack of quantification, it is suggested to provide calibrators with these assays as arbitrary units (AU) only. In this instance, these assays would then be able to detect differences in FH levels between experimental groups (e.g., healthy *vs* disease), as long as the same assay is used for all analyzed groups. In the end, the fact that absolute FH levels vary greatly between assays may not be surprising as calibrators from different sources were used in their calibrations.

For the future, we therefore recommend that a uniform protocol is used regarding sample collection, pre-analytical sample handling and storage. For the assessment itself, it is strongly recommended that standardized assays with a uniform calibrator are used. When antibodies are used for quantification, these must be characterized regarding specificity and cross-reactivity with other proteins (see also below in the next section). In this way, results can be produced that are robustly comparable between different studies.

### Unmet Needs for Validated Tools

In 2016, Nature conducted a survey to understand scientist’s view on the lack of reproducibility in research (124). When asked about the cause, a proportion pointed towards poorly-characterized tools leading to ambiguous findings, which results in an unstable knowledge foundation that is then built upon. Since this survey, different guidelines for *in vitro* and *in vivo* research have been suggested, issued and published (125, 126). Characterization of antibodies as well as validation of tools to quantify proteins is vital for every field, but particularly for the FH protein family considering the high risk of cross-reactivity due to their homology. Yet, when looking at the specific antibodies that are currently used in peer-reviewed publications, results on validation of antibodies are not always provided (**Table 3**). Moreover, whenever present, results on antibody validation are usually in the supplementary data, a scientific habit that perhaps should be reconsidered. Safeguarding accurate antibody validation should be a main concern for all scientists, clinical end-users, industry, journal publishers and antibody-linked research alike. Every researcher has experienced research antibodies that don’t live up to promises or expectations. Either because the antibody does not recognize the desired target, or because they recognize a different protein instead of, or as well as the desired target. Additional problems include functionality of the antibody being limited to certain applications. Too often the choice of an antibody is driven by the number of citations it has in the literature, as scientists falsely assume the antibody must have been validated previously, enabling self-perpetuating artefacts. In this regard, we offer a consensus report by the authors and hope to ignite further discussion among the community to establish recommendations for best practices to improve the reproducibility, validity and to help advance research into the FH-protein family. Obviously, these recommendations are not aimed at antibodies used for mere *in vitro* experiments with purified components, but rather for observational and intervention studies with a large sample size. First of all, it would be advised to (i) reference and (ii) validate the specific antibodies used for the FH protein family research field as detailed as possible (130). We further suggest a best practice protocol for the validation of all detection tools. Based on previous guidelines, we suggest the following 4-step validation protocol for antibodies against the FH protein family (131–134):

**Table 3:** Published antibodies that have been proposed to be specific for each of the Factor H-related proteins.

	Characteristics	Validation steps	Use	Source	Ref.
**FHL-1**	Rabbit pAb IgG anti-human FHL-1	- Direct ELISA for FH and FHL-1. - Double staining with specific FH antibody as well as FH/FHL-1 antibody. - Preincubation of the pAb with FHL-1 prior to IHC - WB with recombinant FH and FHL-1 as well as tissue.	IHC, ELISA, WB.	Non-commercial	(127)
**FHR-1**	Mouse mAb anti-human FHR-1 (Clone JHD10)	- Preincubation of the mAb with FHR-1 prior to IHC. - IHC on material of patients with a combined CFHR1/3 deletion.	IHC, FC, WB.	Non-commercial	(74, 91)
	Mouse mAb anti-human FHR-1	- IHC on material of patients with a homozygous CFHR-1 deficiency.	IHC	(#3078-M01; Abnova, Taipei, Taiwan)	(128, 129)
**FHR-2**	Mouse mAb anti-human FHR-2	Unknown	IF	Non-commercial	(99)
**FHR-3**	Mouse IgG2 mAb anti-human FHR-3 (Clone: RETC-2)	- Direct ELISA for recombinant FH and all FHRs. - WB with recombinant FH, all FHRs and CFHR-3 deficient as well as normal serum. - Mass spectrometry analyses of the immuno-precipitation by the mAb.	IHC, ELISA, WB.	Non-commercial	(110)
	Mouse mAb anti-human FHR3 (Clone: HSL1)	- WB with normal human serum and CFHR3 deficient serum. - Direct ELISA for recombinant FH and all FHRs.	ELISA, FC, WB.	Non-commercial	(80)
**FHR-4**	Mouse IgG1 mAb anti-human FHR-4 (Clone 4.4)	- WB with recombinant FH and FHRs - WB with normal human serum and CFHR3 as well as CFHR4 deficient serum. - WB of the immunoprecipitation by the mAb.	ELISA, WB.	Non-commercial	(111)
	Mouse IgG mAb anti-human FHR-4 (Clone 4E9)	- WB with recombinant FHR-4 and normal human serum	ELISA, WB.	Non-commercial	(28)
	Mouse IgG mAb anti-human FHR-4 (clone 17)	- WB with recombinant FHR-4 and normal human serum	ELISA, WB.	Non-commercial	(28)
	Mouse IgG mAb anti-human FHR-4 (Clone 150)	- Preincubation of the mAb with FHR-4, FHL-1 prior to IHC.	IHC, WB.	Non-commercial	(28)
**FHR-5**	Rabbit pAb IgG anti-human FHR-5	- Preincubation of the pAb with FHR-5 prior to IHC. - Direct ELISA for C3c, iC3b and C3d with and without FHR-5.	IHC, ELISA.	(#81494-D01P; Abnova, Taipei, Taiwan)	(128, 129)
	Mouse mAb anti-human FHR-5 (Clone K2.254)	Unknown	IHC, ELISA	Non-commercial	(55)
	Mouse mAb IgG1 anti-human FHR-5	Unknown	IHC, ELISA, WB, FC.	(#390513, R&D Systems)	(99)
	Mouse mAb IgG1 anti-human FHR-5 (clone 5.1)	- WB with recombinant FH and FHRs - Direct ELISA for recombinant FH and all FHRs.	ELISA, WB	Non-commercial	(37)
	Mouse mAb IgG1 anti-human FHR-5 (Clone 5.4)	- WB with recombinant FH and FHRs - Direct ELISA for recombinant FH and all FHRs.	ELISA, WB	Non-commercial	(37)

An overview of the published antibodies that are proposed to be specific for one of the Factor H-related proteins. Specific information is provided for each antibody in regards to their characteristics, validation steps, application, source and the reference.

ELISA, enzyme-linked immunoassay; FC, flow cytometry; FH, Factor H; FHL-1. Factor H-like 1; FHR, Factor H-related protein; IF, immunofluorescence; IgG, immunoglobulin; IHC, immunohistochemistry; mAb, monoclonal antibody; pAb, polyclonal antibody; WB, Western blot.

1. Show whole Western blot of target human/animals samples detected with the antibody.

Value: Knowledge regarding the respective protein size(s).

Cave: Only applicable if the antibody is reactive in Western blot.

2. Compare binding pattern of the antibody for ALL the different known FH-protein family members (e.g. ELISA, Western blot or Dot blot).

Value: Characterization of intra-protein family specificity.

Cave: Access to validation material can be restricted.

3. Test the antibody reactivity against either a.) non-depleted vs FH-protein family members depleted human serum/plasma (if available), b.) non-deficient cells vs target gene deficient cells (if available) or c.) wildtype vs knockout animals (e.g., ELISA, Western Blot or immunohistochemistry, if available).

Value: Species specificity of the antibody can be determined independent from unknown protein modifications.

Cave: Access to validation material can be restricted.

4. Characterize the specificity of an antibody using immunoprecipitation with subsequent mass spectrometry analysis from the respective target tissue.

Value: Tissue specific cross reactions of the whole antibody (even the Fc-part) will be deciphered.

Cave: Specialized collaborating laboratories are needed.

It would be preferred to perform this 4-step validation protocol in line with an already validated, antibody as a quality control (if available). A single protocol will not match all applications and researchers must ensure that the reported validation holds true for their specific use. Overall, we are convinced that validation of tools, together with transdisciplinary collaboration and transparency within the community will enable research to move forward and get one step closer towards deciphering the mode of action of the FH-protein family.

### Limitations of Animal Models

Murine models have demonstrated high value for establishing fundamental principles of complement biology (135). The murine version of FH (mFH) was first identified in 1986 and is very similar in structure and function to the human FH (136). As its human equivalent, mFH has a molecular weight of around 155 kDa, has several glycosylation sites, is positioned on chromosome 1 and is primarily produced by the liver. The plasma concentration of mFH is estimated to be similar to human FH, but an exact concentration of mFH has not been determined yet (135, 136). Clear differences exist as well between mFH and human FH. For instance, in contrast to the human *CFH* gene, the *mCFH* gene does not have an alternative splicing variant and thus no murine equivalent of FHL-1 has been identified. Overall, The *mCFH* gene shares 63% of sequence identity with the human *CFH* gene (135). Despite the differences in genes and protein structures across mouse and human, it has been observed repeatedly that essential activation and regulatory functions of this system are retained across species. In accordance, genetically deficient animals have provided a powerful tool to help understand the function of FH.

The link between FH and disease is older than is frequently reported. Høgåsen et al. described more than 25 years ago that a hereditary deficiency of FH in pigs consistently led to the development of lethal renal disease, namely C3G (137). These findings were later confirmed in rodent models, demonstrating that mice deficient in FH had uncontrolled complement activation resulting in C3G (138). Interestingly, later studies revealed that aged *CFH^-/-^* mice also develop retinal abnormalities and visual dysfunction, resembling AMD (139). The broad outlines are therefore clear, dysfunction of FH leads to uncontrolled complement activation resulting in tissue injury and thus causing disease. However, it was not clear what then determines whether defects in FH cause one specific disease but not the other. Animal models have helped to attribute the different functions of FH to specific domains within the protein, and thereby reveal specific genotype–phenotype connections in FH that lead to either complement-mediated thrombotic microangiopathy (TMA) or C3G and AMD. A study by Pickering et al. uncovered that a homozygous FH deficiency in mice leads to defective function of FH in the fluid-phase triggering the development of C3G and AMD (138). In contrast, loss of the SCR 16-20 region of FH impairs the ability of FH to control complement activation on surfaces, causing spontaneous TMA (60). In conclusion, the use of animal models has helped significantly to understand the function of complement proteins and their role in disease, especially for FH.

Animal models have barely been used to study the other members of the Factor H family. As mentioned before, no murine equivalent for the human FHL-1 exists. Furthermore, the *CFHR* genes have arisen during evolution through duplication events of the *CFH* gene (12). Extensive analysis of the human *CFH* and *CFHR* gene loci using Alu/L1 repeat dating established that these duplication events occurred after the separation of rodent and primate lineages and therefore no FHR orthologues exist in mice. More specifically, the *mCFHR* genes differ in structure, domain composition, and sequence from the human genes (32). Like the human FHR proteins, a total of five murine *CFHR* genes have been suggested (*mCFHR-A*, *mCFHR-B*, *mCFHR-C*, *mCFHR-D* and *mCFHR-E*) and evidence for four mFHR proteins (FHR-B, FHR-C, FHR-D and FHR-E) has been derived from mRNA transcripts isolated from mouse liver (48, 140–143). However, altogether, this suggests that direct comparisons between the human and mouse FHR proteins is not informative and, therefore, any mouse FHR homologs need to be identified, if they exist, by functional studies before rodent models can be used to further study the role of the FH protein family in human health and disease. Alternatively, genetic engineering approaches could be used to create a set of humanized transgenic mice to more closely mimic the human FHR situation. Until that is achieved, the lack of animal models remains a major barrier hindering the elucidation of disease mechanisms and drug development. More importantly, the absence of appropriate animal models stresses the importance of appropriate human assays to correctly identify and study the Factor H protein family in humans.

## Therapeutic Value of FH and Derivatives in Complement-Mediated Diseases

The various disorders linked to the FH family tend to be difficult to treat and some are even incurable. An obvious therapeutic strategy for these diseases could therefore be the administration of (purified or recombinant) FH to restore complement regulation. Indeed, both *in vitro* and animal studies have demonstrated the therapeutic value of FH (63, 137, 144–146). In *CFH^-/-^* pigs, a single dose of 5 mg/kg porcine FH resulted in normalization of plasma C3 levels and diminished systemic complement activation for almost 3 days (137). In *CFH^-/-^* mice, both purified mouse and purified human FH led to a rapid increase of plasma C3 levels and resolution of renal C3 deposition (144, 146). However, FH supplementation as a therapy would require large amounts of biologically active protein due to high circulating levels in healthy individual, making it labor and cost intensive. Various strategies have been tested to resolve these problems. Several groups have demonstrated successful production of high yields of recombinant FH in different expression systems (such as yeast and moss) as an alternative and economically viable method (147, 148). Others have created derivates or fusion proteins from FH with enhanced pharmacokinetic and pharmacodynamic properties. Smaller constructs of FH have been created by combining the regulatory domains (N-terminus) with the surface-recognition domains (C-terminus) (121, 149, 150, 158). These FH constructs can regulate complement *in vivo*, and effectively reverse renal C3 deposition and restore plasma C3 levels in *CFH^-/-^* mice. However, the short half-life of these constructs remains an important limitation. FH fusion protein have also been engineered as a therapeutic approach (151–153). Most extensively studied is the CR2-FH fusion protein, that links the C3d binding domain of complement receptor 2 (CR2) to the complement inhibitory domain of FH, thus ensuring targeted regulation by FH at sites of complement activation (154). Treatment with CR2-FH was beneficial in animal models of eye, kidney and autoimmune diseases (155–157). Finally, local injection of FH (or derivates) is another approach to circumvent the need for large amounts of biologically active protein. A clinical trial investigating the safety and effectivity of recombinant FH (GEM103) administered through intravitreal injections for the treatment of geographic atrophy secondary to dry AMD is currently on-going (ClinicalTrials.gov identifier, NCT04246866).

## Future Perspective

A multidisciplinary approach is mandatory to overcome the challenges mentioned above, and is only possible through interdisciplinary collaboration between biologists, chemists, geneticists and physicians. But, where to start? As suggested by the quote of William Thomson, the authors of this paper believe that we should essentially begin with quantifying the levels and activity of the different members of the FH-family in health and disease. Detection of the FH-protein family will enable the scientific and clinical community to advance our understanding of the role of the FH-protein family in infectious, eye, kidney and autoimmune diseases, and potentially help treat these disorders.

## Conclusion

As described, the FH-family, consists of FH, FHL-1 and the five FHR proteins which are important regulators of the complement system. Mutations and polymorphisms in the FH-family are involved in several diseases, indicating a potential crucial role of the FH-family in both health and disease. However, diagnosis and therapy of these partially incurable pathologies is to-date not related to the FH-protein family, due to a lack of fundamental knowledge of (i) the molecular mechanisms leading to disease, (ii) unknown functional, convincing principles of FH-protein family members, (iii) absent standardized diagnostics and (iv) missing suitable drug candidates. To overcome these challenges, an ardent multidisciplinary approach is required through interdisciplinary collaboration.

## Author Contributions

All authors contributed equally and the review was written together. All authors critically reviewed the manuscript prior to submission.

## Funding

This project has received funding from the European Union’s Horizon 2020 research and innovation programme under grant agreement No 899163.

## Conflict of Interest

MS was employed by Microcoat Biotechnologie GmbH. ET was employed by Hycult Biotech.

The remaining authors declare that the research was conducted in the absence of any commercial or financial relationships that could be construed as a potential conflict of interest.

## Abbreviations

aHUS, Atypical hemolytic uremic syndrome; AMD, Age-related macular degeneration; ANCA, Anti-neutrophil cytoplasmic antibody; AP, Alternative pathway; AU, Arbitrary units; BLAST, Basic local alignment search tool; C3G, C3 glomerulopathy; CCP, Complement control protein; *CFH*, Complement Factor H gene; *CFHR*, Complement Factor H-related genes; CP, Classical pathway; CRP, C-reactive protein; FB, Factor B; FH, Factor H; FHL-1, Factor H-like 1; FHR, Factor H-related protein; FI, Factor I; FISH, Fluorescence *in situ* hybridization; IgAN, Immunoglobulin A nephropathy; LP, Lectin pathway; mAb, Monoclonal antibody; mCFH, Murine complement Factor H gene; mCFHR, Murine complement Factor H-related gene; mFH, Murine Factor H; mFHR, Murine Factor H-related protein; NLRP3, NOD-, LRR- and pyrin domain-containing protein 3; PTX3, Pentraxin-3; RCA, Regulators of complement activation gene cluster; SLE, Systemic lupus erythematosus; TMA, Thrombotic microangiopathy.
