# Corrigendum: A Family Affair: Addressing the Challenges of Factor H and the Related Proteins

**DOI:** 10.3389/fimmu.2022.937609

**Published:** 2022-05-30

**Authors:** Felix Poppelaars, Elena Goicoechea de Jorge, Ilse Jongerius, Antje J. Baeumner, Mark-Steven Steiner, Mihály Józsi, Erik J. M. Toonen, Diana Pauly, the SciFiMed consortium

**Affiliations:** ^1^ Department of Internal Medicine, Division of Nephrology, University Medical Center Groningen, University of Groningen, Groningen, Netherlands; ^2^ Department of Immunology, Faculty of Medicine, Complutense University and Research Institute Hospital 12 de Octubre (imas12), Madrid, Spain; ^3^ Department of Immunopathology, Sanquin Research and Landsteiner Laboratory of the Academic Medical Centre, University of Amsterdam, Amsterdam, Netherlands; ^4^ Department of Pediatric Immunology, Rheumatology, and Infectious Diseases, Emma Children’s Hospital, Amsterdam University Medical Centre, Amsterdam, Netherlands; ^5^ Institute of Analytical Chemistry, Chemo-and Biosensors, Faculty of Chemistry and Pharmacy, University of Regensburg, Regensburg, Germany; ^6^ Microcoat Biotechnologie GmbH, Bernried am Starnberger See, Germany; ^7^ Department of Immunology, ELTE Eötvös Loránd University, Budapest, Hungary; ^8^ MTA-ELTE Complement Research Group, Eötvös Loránd Research Network (ELKH), Department of Immunology, ELTE Eötvös Loránd University, Budapest, Hungary; ^9^ R&D Department, Hycult Biotech, Uden, Netherlands; ^10^ Department of Ophthalmology, University Hospital Regensburg, Regensburg, Germany; ^11^ Experimental Ophthalmology, University Marburg, Marburg, Germany

**Keywords:** complement system, factor H (FH), factor H-related protein, factor H-like protein 1, challenges - development directions

There is an error in the **Funding** statement as published. The correct name for the Funder is “**the European Union’s Horizon 2020 research and innovation programme**”. The correct Funding statement is “This project has received funding from the European Union’s Horizon 2020 research and innovation programme under grant agreement No 899163”.

The authors apologize for this error and state that this does not change the scientific conclusions of the article in any way. The original article has been updated.

## Publisher’s Note

All claims expressed in this article are solely those of the authors and do not necessarily represent those of their affiliated organizations, or those of the publisher, the editors and the reviewers. Any product that may be evaluated in this article, or claim that may be made by its manufacturer, is not guaranteed or endorsed by the publisher.

